# Inflammation and Tissue Remodeling in the Bladder and Urethra in Feline Interstitial Cystitis

**DOI:** 10.3389/fnsys.2018.00013

**Published:** 2018-04-13

**Authors:** F. Aura Kullmann, Bronagh M. McDonnell, Amanda S. Wolf-Johnston, Andrew M. Lynn, Daniel Giglio, Samuel E. Getchell, Wily G. Ruiz, Irina V. Zabbarova, Youko Ikeda, Anthony J. Kanai, James R. Roppolo, Sheldon I. Bastacky, Gerard Apodaca, C. A. Tony Buffington, Lori A. Birder

**Affiliations:** ^1^Department of Medicine, School of Medicine, University of Pittsburgh, Pittsburgh, PA, United States; ^2^Department of Pharmacology, The Sahlgrenska Academy, Göteborg University, Göteborg, Sweden; ^3^Department of Pharmacology and Chemical Biology, School of Medicine, University of Pittsburgh, Pittsburgh, PA, United States; ^4^Department of Pathology, School of Medicine, University of Pittsburgh, Pittsburgh, PA, United States; ^5^Department of Medicine and Epidemiology, University of California, Davis, Davis, CA, United States; ^6^Department of Veterinary Clinical Sciences, The Ohio State University, Columbus, OH, United States

**Keywords:** bladder, urethra, paraneurons, urothelium, von Brunn’s nest

## Abstract

Interstitial cystitis/bladder pain syndrome (IC/BPS) is a debilitating chronic disease of unknown etiology. A naturally occurring disease termed feline interstitial cystitis (FIC) reproduces many features of IC/BPS patients. To gain insights into mechanisms underlying IC/BPS, we investigated pathological changes in the lamina propria (LP) of the bladder and proximal urethra in cats with FIC, using histological and molecular methods. Compared to control cat tissue, we found an increased number of de-granulated mast cells, accumulation of leukocytes, increased cyclooxygenase (COX)-1 expression in the bladder LP, and increased COX-2 expression in the urethra LP from cats with FIC. We also found increased suburothelial proliferation, evidenced by mucosal von Brunn’s nests, neovascularization and alterations in elastin content. Scanning electron microscopy revealed normal appearance of the superficial urethral epithelium, including the neuroendocrine cells (termed paraneurons), in FIC urethrae. Together, these histological findings suggest the presence of chronic inflammation of unknown origin leading to tissue remodeling. Since the mucosa functions as part of a “sensory network” and urothelial cells, nerves and other cells in the LP are influenced by the composition of the underlying tissues including the vasculature, the changes observed in the present study may alter the communication of sensory information between different cellular components. This type of mucosal signaling can also extend to the urethra, where recent evidence has revealed that the urethral epithelium is likely to be part of a signaling system involving paraneurons and sensory nerves. Taken together, our data suggest a more prominent role for chronic inflammation and tissue remodeling than previously thought, which may result in alterations in mucosal signaling within the urinary bladder and proximal urethra that may contribute to altered sensations and pain in cats and humans with this syndrome.

## Introduction

Interstitial cystitis/bladder pain syndrome (IC/BPS) is a chronic pelvic pain syndrome characterized by suprapubic pain, and urinary frequency and urgency in the absence of an alternative explanation for the symptoms (Hanno et al., 2011). Despite years of intense research, the etiology remains obscure and therapies remain scarce due to incomplete understanding of pathophysiology and mechanisms that mediate this dysfunction as well as lack of relevant animal models that represent the complexity of the naturally occurring syndrome. Additionally, the diagnosis is very difficult, with some studies even suggesting two separate disorders, one associated with chronic inflammation (IC) and one lacking an inflammatory component (BPS; Leiby et al., 2007; Payne, 2017).

Feline interstitial cystitis (FIC) is a naturally occurring idiopathic condition of domestic cats that exhibits physiological and pathological changes similar to those associated with the non-ulcerative form of IC/BPS (Buffington et al., 1999). Previous studies revealed urothelial thinning and desquamation of bladder tissue from cats with FIC (Lavelle et al., 2000; Hauser et al., 2015), similar to reports from patients with IC/BPS (Lynes et al., 1990). A number of alterations in the expression of urothelial-associated proteins (e.g., zonula occludens type 1, E-cadherin, and uroplakins; Lavelle et al., 2000; Slobodov et al., 2004; Hauser et al., 2008, 2015), receptors/ion channels (e.g., purinergic; transient receptor potential channels—TRPs), and release of transmitters (e.g., ATP, NO) from the urothelium, both basal or in response to mechanical or chemical stimuli were also identified (Birder et al., 2003, 2004, 2005; Sun and Chai, 2004; Kumar et al., 2007). These alterations can contribute to the disruption of urothelial barrier and sensory functions, resulting in increased afferent nerve activity and amplified input to the CNS (Birder et al., 2004, 2010; Sun and Chai, 2004; Roppolo et al., 2005; Kumar et al., 2007), and may represent underlying mechanisms for bladder symptoms such as hypersensitivity, pain or urgency.

In addition to changes in the urothelium, studies have shown alterations in the lamina propria (LP), an area thought to be an integration center of sensory and motor pathways. Bladder biopsies from IC/BPS patients have shown increased mast cell count, alterations in interstitial cell-like populations, presence of inflammatory infiltrates, pyuria, edema, patchy fibrosis and vascular lesions consisting of damaged endothelial cells (Lynes et al., 1990; Elbadawi and Light, 1996; Tomaszewski et al., 2001; Gevaert et al., 2011). Most of these alterations are consistent with chronic mucosa inflammation and correlate with voiding symptoms and pain, suggesting that disruption of signaling in the LP plays a role in IC/BPS.

While much of research has focused on the bladder itself as a cause of IC/BPS, significantly less attention has focused on the proximal urethra, a region shown to play a significant role in bladder function. In animals and humans, sensory input from the urethra, such as flow of fluid, distention, irritation or electrical stimulation, modulates bladder function and visceral pain (Jung et al., 1999; Robain et al., 2001; Shafik et al., 2003a,b; Deckmann et al., 2014; Danziger and Grill, 2016). Anatomically, the proximal urethra is densely innervated by sensory nerves positive for calcitonin gene related peptide (CGRP) and substance P (SP; Barry et al., 2017; Kullmann et al., 2018), and contains specialized cells embedded within the epithelium that are likely to be involved in the detection, processing and transmission of sensory information. Among these are the paraneurons (also termed brush-cells or neuroendocrine cells). Several populations of paraneurons expressing a number of mediators such as acetylcholine (ACh), serotonin (5-HT) and somatostatin are present in the urethral epithelium (Vittoria et al., 1990; Deckmann et al., 2014; Kullmann et al., 2018). Although their function is not well understood, it is thought that upon detection of mechanical and chemical stimuli, paraneurons may release transmitters (5-HT, ACh) to activate nearby sensory nerves (Deckmann et al., 2014; Kullmann et al., 2018). It is conceivable that changes in the properties and/or morphology of the proximal urethra, including the paraneurons, can impact sensory information processing and contribute to the symptoms of bladder dysfunction in IC/BPS.

Given that the urothelium and LP are major sites of integration of sensory signaling, and that abnormal sensory processing is a hallmark of IC/BPS, we investigated morphological changes in the bladder and urethral epithelium and LP from cats with FIC.

## Materials and Methods

### Animals

**Figure 1:**
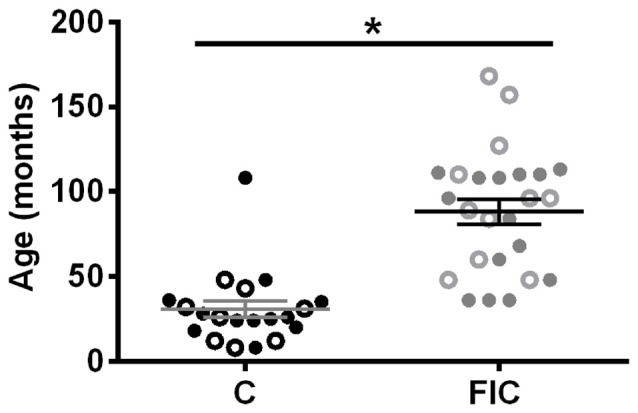
Age and sex distribution of control (C) and feline interstitial cystitis (FIC) cats. Open circle symbols indicate males, closed circle symbols indicate females. Statistical significance was tested using unpaired *t*-test. Asterisk (*) indicates *p* < 0.05.

This study was carried out in accordance with the recommendations and approval of the Institutional Animal Care and Use Committee at the University of Pittsburgh and the Ohio State University. Cats with FIC were obtained as donations from clients (total 26 cats: 11 males, 15 females, age 36–168 months; Figure 1), and diagnosed at the Ohio State University Veterinary Teaching Hospital, using established criteria (Buffington et al., 1999). The control group (total 22 cats: eight males, 12 females, two unknown, age 8–108 months; Figure 1; Liberty Research Inc., Waverly, NY, USA) was assessed using the same criteria. Tissue was collected from deeply anesthetized cats (5% isoflurane). Anesthesia depth was determined by the absence of the withdrawal reflex to a strong pinch of the hind paw and absence of an eye blink reflex to tactile stimulation of the cornea. The bladder and urethra were removed and placed in PBS, opened longitudinally (dome to urethra) and separated at the bladder neck, defined as ~0.7–1 cm distal to the ureteric orifices. The bladder and proximal urethra (defined as ~1.5 cm distal from the bladder neck) were divided into smaller sections of approximately 0.5–1.5 × 0.5–1.5 cm and prepared according to the methods described below. Previous studies have established that the size of these tissue samples is larger than the distances between affected and unaffected areas in the bladder urothelium in FIC cats and thus considered appropriate for detecting possible alterations (Lavelle et al., 2000; Hauser et al., 2015). Some assays required a larger sample of tissue than others, thus in some instances tissue from one animal was used only for a limited number of assays. Western blot assay was performed using a longitudinal bladder strip that contained bladder dome, mid and neck. Histology was performed using samples mostly from the mid bladder. All urethra samples were from the proximal urethra. After tissue collection cats were euthanized with an overdose of sodium pentobarbital.

### Scanning Electron Microscopy

Urethral tissue from three control (one female, two males) and three FIC (two females, one male) cats was fixed by immersion (90 min, room temperature) in 2.0% (vol/vol) glutaraldehyde, 2.0% (wt/vol) paraformaldehyde in 200 mM sodium cacodylate buffer, pH 7.4, containing 1 mM CaCl_2_, and 0.5 mM MgCl_2_. Tissue was washed with 100 mM sodium cacodylate, postfixed in 1.0% w/v OsO_4_, 1.0% w/v K_4_Fe (CN)_ 6_, in 100 mM sodium cacodylate, pH 7.4, then dehydrated for 15 min each in a graded series of ethanol: 30%, 50%, 70%, 95%, 100%. The dehydrated samples were critical point dried, sputter coated with gold-palladium, and viewed using a JEOL model JSM T-300 scanning electron microscope at 20 kV. Samples were photographed using an attached Nikon D40 digital camera. Digital images were imported into Photoshop CS6 (Adobe Systems, San Jose, CA, USA) and contrast and brightness corrected. Composite images were assembled using Adobe Illustrator CS6.

### Histology

Bladder and urethra tissues were fixed in 10% formalin, embedded in paraffin and cut at 4 μm thickness. Slides were stained with toluidine blue for mast cell and neutrophil detection, hematoxylin and eosin or Masson’s trichrome for tissue evaluation, and Verhoeff-Van Gieson for elastin. All procedures were performed by the University of Pittsburgh Department of Pathology technicians using routine staining protocols.

### Image Acquisition and Data Analysis

Images were taken with a BX-62 Olympus upright microscope equipped with 10× 0.4 NA, 20× 0.7 NA air objectives or 40× 1.0 NA oil objective using HCImage software (Hamamatsu Photonics, Bridgewater, NJ, USA). Assessments were conducted by 1–3 blinded researchers and data averaged. For mast cell count, images of toluidine blue stained slides were taken with a 20× objective in a grid-like sequence of 6–30 images per tissue (depending on the size of available tissue), containing the urothelium, LP and smooth muscle (SM). These images were combined into a single mosaic image using Microsoft Image Composite Editor software and analyzed using ImageJ. Mast cells, both granulated and de-granulated were counted in the LP and SM by three blinded observers and counts averaged. Immune cell infiltration (or leukocyte accumulation) was defined as a group of dense cells containing, among other cells types, neutrophils based on the presence of small dark cytoplasmic vesicles. After visual inspection of the toluidine blue stained slides, two 20× pictures of representative areas were taken from each tissue. Quantification of leukocyte accumulation was assessed by circling the groups of cells and computing the area using ImageJ. Slides that did not have accumulations received a 0 for the area. A histogram was then plotted for comparing control and FIC tissue. Blood vessels, identified by the pattern of endothelial cells, were counted in LP and data presented as number of vessels per area of tissue. Elastin fibers in the LP were assessed using the Verhoeff-Van Gieson stained tissue. The density of fibers was first evaluated on a scale from 0 to 5 (0 no fibers, 5 dense fibers) by a blinded observer. Using this visual scale, elastin content in both bladder and urethra LP was higher in FIC than in control tissue (bladder control: 1.7 ± 0.4, *n* = 15; bladder FIC 3.0 ± 0.5, *n* = 14; urethra control: 1.3 ± 0.6, *n* = 3; urethra FIC 3.7 ± 0.5, *n* = 7). Fibers were then quantified by another blinded observer by counting the number of fibers and measuring their length in two random LP areas of approximately 50–100 × 50–100 μm each per tissue. Data from these two areas were averaged and taken as one measurement per tissue. The presence or absence of von Brunn’s nests was determined by visual inspection of the H&E stained slides.

### Western Blot Immunoblotting

Epithelium with LP (collectively termed mucosa) was surgically separated from the SM. Mucosa was homogenized using Lysing Matrix D in a FastPrep 24 instrument (MP Biomedicals, Solon, OH, USA) in HBSS (in mM: KCl 5, KH_2_PO_4_ 0.3, NaCl 138, NaHO_3_ 4, Na_2_HCO_3_ 0.3, Na_2_HPO_4_ 0.3, glucose 5.6, Hepes 10; pH 7.4) containing complete protease inhibitor cocktail (1 tablet/10 ml, Roche, Indianapolis, IN, USA) and phosphatase inhibitor cocktail (Sigma, 1:100). After centrifugation (13,000 rpm; 15 min at 4°C), the membrane protein fraction was prepared by suspending the membrane pellets in lysis buffer containing 0.3 M NaCl, 50 mM Tris-HCl (pH 7.6) and 0.5% Triton X-100 and the same concentration of protease inhibitors as above. The suspensions were incubated on ice and centrifuged. Protein concentrations of the combined supernatants were determined using the Pierce BCA protein assay (Thermo Scientific, Rockford, IL, USA). After denaturation (100°C for 5 min) in the presence of Laemmli sample buffer, lysate from each sample was separated on 4%–15% TGX Stain-Free SDS-PAGE gel (Bio-Rad Laboratories, Hercules, CA, USA). Following transfer of proteins to polyvinylidene fluoride membranes (Bio-Rad), the membranes were incubated in 5% (w/v) milk TBS-T (20 mM Trizma, 137 mM NaCl, 0.1% Tween-20, pH 7.6), rinsed with TBS-T, and incubated overnight at 4°C with primary antibody against cyclooxygenase (COX)-1 (1:1000; Cayman Chemical, Cat #160108, RRID:AB_10078457) and COX-2 (1:1000; Cayman Chemical, Cat # 160106, RRID:AB_10077935) in 5% milk TBS-T or 5% BSA TBS-T. After washing in TBS-T, the membranes were incubated with secondary antibodies donkey anti-rabbit HRP (1:1000; GE Healthcare Biosciences, Marlborough, MA, USA) for 1 h in 5% milk TBS-T, washed, incubated in WesternBright Quantum or Sirius (Advansta, Menlo Park, CA, USA) and exposed to film. As a loading control, total protein per sample was determined using Bio-Rad Stain Free SDS-PAGE gel technology in both the gel and the membrane after transfer. UV-activated protein fluorescence was imaged using a ChemiDoc MP (Bio-Rad). The volume (intensity) of each protein species was determined and normalized to total protein stain using Image Lab software (Bio-Rad). Samples from control cats and cats with FIC were assayed in parallel in western blots. However, due to the large sample size, multiple blots were performed. Because western data is qualitative and dependent on exposure time, each set of data from each blot was normalized by calculating the fold change per sample compared to the average control for that particular blot. Data are expressed as fold change relative to the control.

### Statistical Analysis

Results are expressed as mean ± SEM. Values for control vs. FIC were tested with Student’s *t*-test, non-parametric Mann-Whitney test, Chi-square test, or Kolmogorov-Smirnov test as indicated in the figure legends, using GraphPad Prism 6 (GraphPad Software, La Jolla, CA, USA). Statistical significance was considered at *p* < 0.05.

## Results

All cats used in this study were diagnosed at the Ohio State University Veterinary Teaching Hospital using established criteria (Buffington et al., 1999; Buffington, 2004). Cats with FIC were significantly older than control cats (Figure 1) and exhibited urinary dysfunction characterized by frequent urination, visceral pain and hypersensitivity, with waxing and waning symptoms (Buffington, 2011).

### Inflammatory Markers, Tissue Remodeling and Neovascularization in FIC Tissue

**Figure 2:**
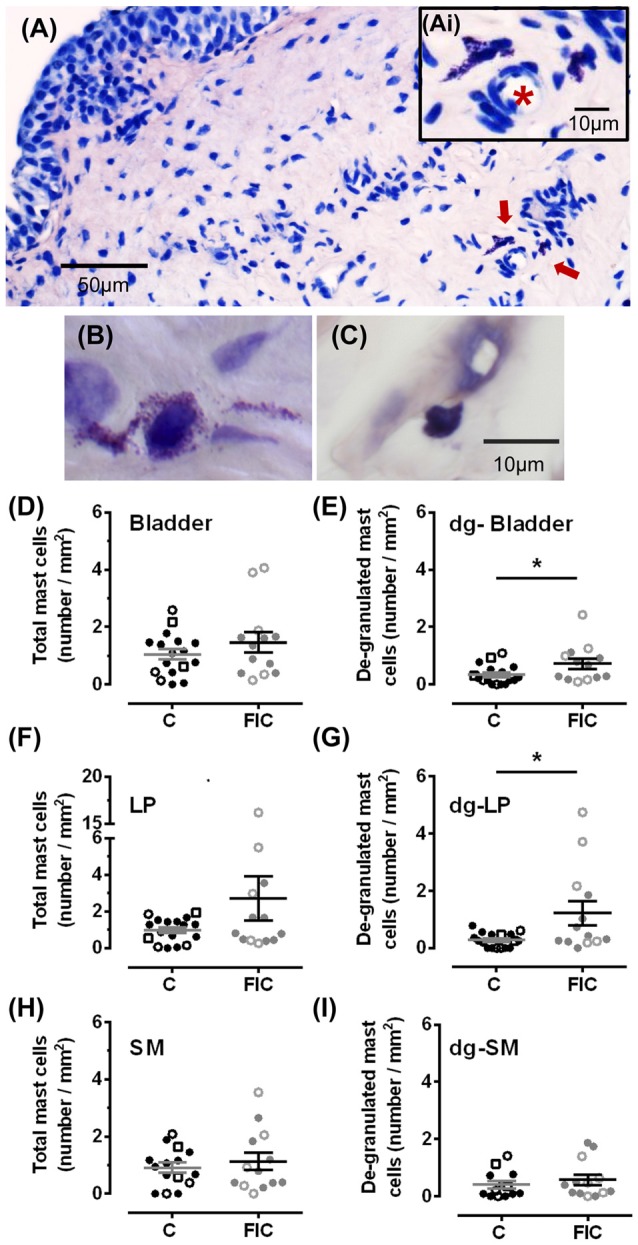
Mast cell quantification in bladder tissue from control and FIC cats. **(A)** Example of de-granulated mast cells, (red arrows) from a FIC cat. **(Ai)** Inset shows higher magnification of the area indicated by the red arrows and illustrates the presence of de-granulated mast cells in the vicinity of a blood vessel (red star placed in the lumen of the blood vessel). **(B,C)** Examples of de-granulated **(B)** and not de-granulated **(C)** mast cells from an FIC cat. **(D–I)** Quantification of mast cells and de-granulated (dg-) mast cells in bladder tissue from control (*n* = 17; individual data points shown in black, and average ± SEM in gray) and FIC (*n* = 13; individual data points shown in gray, and average ± SEM in black) cats. Statistical significance was tested using Mann-Whitney test. Asterisk (*) indicates *p* < 0.05. **(D)** Total number of mast cells, including granulated and de-granulated, in bladder tissue which is comprised of lamina propria (LP) and smooth muscle (SM) in control and FIC tissue (*p* = 0.50). **(E)** Total number of de-granulated mast cells in the bladder in control and FIC tissue (*p* = 0.037). **(F)** Total number of mast cells in the LP in control and FIC tissue (*p* = 0.38). **(G)** Total number of de-granulated mast cells in the LP in control and FIC tissue (*p* = 0.01). **(H)** Total number of mast cells in the SM in control and FIC tissue (*p* = 0.90). **(I)** Total number of de-granulated mast cells in the SM in control and FIC tissue (*p* = 0.47). In all panels, each symbol represents data from one animal. Open circles represent males, closed circles females and open squares represent undocumented sex. In **(C)**, the highest data point is from a male FIC cat 48 months old that fit the criteria to be included in the study.

**Figure 3:**
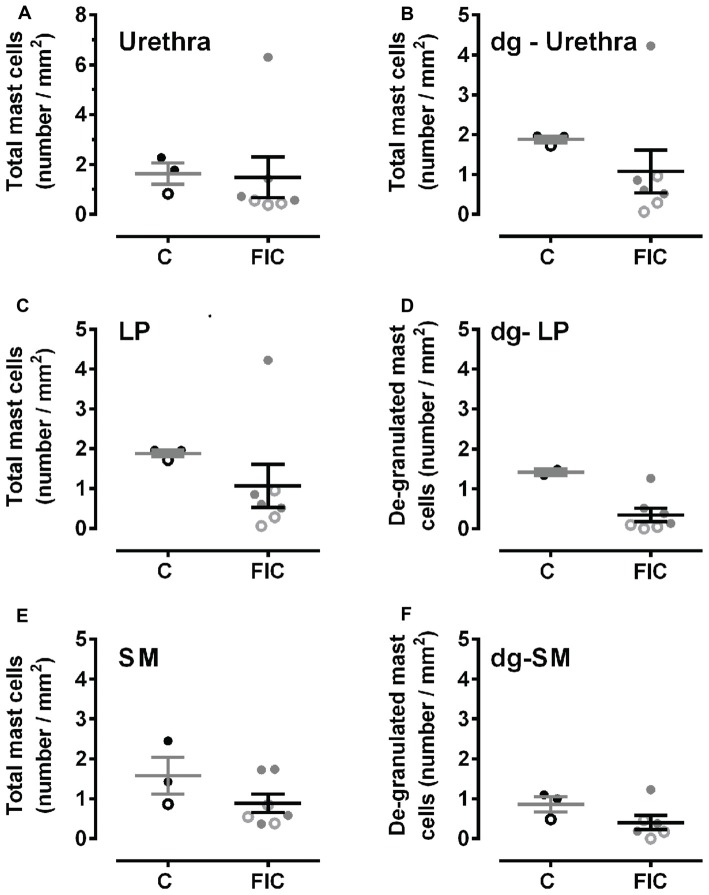
Mast cell quantification in urethral tissue from control and FIC cats. Data are presented as single points from individual cats with mean ± SEM. Control cats (*n* = 3) are shown in black and FIC (*n* = 7) in gray. Statistical significance was tested using Mann-Whitney test. **(A)** Total number of mast cells, including granulated and de-granulated (dg-), in the urethral tissue which is comprised of LP and SM in control and FIC tissue (*p* = 0.18). **(B)** Total number of de-granulated mast cells in the whole urethra in control and FIC tissue (*p* = 0.60). **(C)** Total number of mast cells in the LP in control and FIC tissue (*p* = 0.11). **(D)** Total number of de-granulated mast cells in the LP in control and FIC tissue (*p* = 0.55). **(E)** Total number of mast cells in the SM in control and FIC tissue (*p* = 0.18). **(F)** Total number of de-granulated mast cells in the SM in control and FIC tissue (*p* = 0.16). In all panels, each symbol represents data from one animal. Open circles represent males, closed circles females and open squares represent undocumented sex.

Mast cells are associated with inflammation, and an increase in their number in tissue biopsies has even been used as a diagnostic criterion for IC/BPS in patients (Letourneau et al., 1992, 1996; Theoharides et al., 1995, 2001; Pang et al., 1996; Larsen et al., 2008). To determine whether there was an increase in mast cells in cats with FIC compared to healthy controls, we assessed granulated and de-granulated mast cells in the LP and SM of the bladder and urethral tissue (Figures 2, 3). In the bladder, the number of mast cells per area of tissue in either the LP or the SM, was not significantly higher in the FIC vs. control (Figures 2D,F,H). However, the number of de-granulated cells, especially in the LP, was significantly higher in the FIC bladders (Figures 2E,G). In the urethra, neither the total number of mast cells nor the de-granulated mast cell number was significantly different in either LP or SM, in the FIC vs. control (Figure 3). No significant correlation between age and number of mast cells was observed in either control or FIC cat bladder or urethrae (Pearson correlation r for bladder: −0.25 and −0.20 for control and FIC cats, respectively; for urethra: −0.07 and 0.05 for control and FIC cats, respectively). These results indicate that the bladder LP of cats with FIC contained a large number of de-granulated mast cells, consistent with findings in human tissue (Letourneau et al., 1992, 1996; Theoharides et al., 1995, 2001; Pang et al., 1996; Larsen et al., 2008).

**Figure 4:**
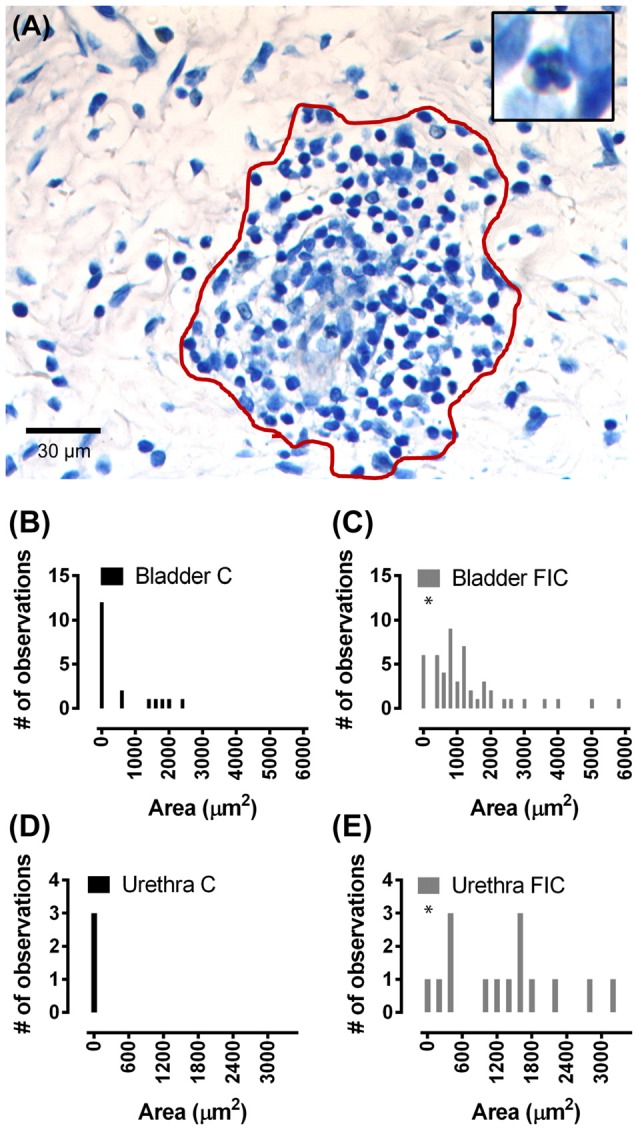
Immune cell infiltration in the LP of the bladder and urethra. **(A)** Example of leukocyte accumulation and area (red line) used for quantification, in the bladder of a FIC cat. Inset illustrates a neutrophil, characterized by the presence of dark small vesicles. **(B,C)** Histogram of area of immune cell infiltrate in the LP of the bladder from control (black; *n* = 17) and FIC (gray; *n* = 13) cats (Kolmogorov-Smirnov test, *p* = 0.02) cats showing only values smaller than 6000 μm^2^. One measurement in control and two measurements in FIC tissue were larger than 6000 μm^2^. Bin size 200 μm^2^. **(D,E)** Histogram of area of immune cell infiltrate in the LP of the urethra of control (black; *n* = 3) and FIC (gray; *n* = 7) cats (Kolmogorov-Smirnov test, *p* = 0.02) showing only values smaller than 3500 μm^2^. Bin size 200 μm^2^. There was no immune cell infiltrate in the urethra of control cats (black bar centered at 0; *n* = 3 cats). Three measurements in FIC tissue were larger than 3500 μm^2^. Statistical significance was tested using Kolmogorov-Smirnov test. Asterisk (*) indicates *p* < 0.05.

Accumulation of leukocytes, consisting of neutrophils identifiable by small dark cytoplasmic vesicles (inset in Figure 4A), and other types of reactive lymphocytes, is another indication of tissue inflammation, previously described in the tissue of IC/BPS patients (Lynes et al., 1990; Tomaszewski et al., 2001; Gamper et al., 2013). In control cat tissue, accumulation of leukocytes in the LP was detected in 29% (5 out of 17) bladders and 0% (0 out of 3) urethrae. In contrast, in FIC cat tissue, accumulation of leukocytes in the LP was detected in 54% (7 out of 13) bladders and 75% (3 out of 4) urethrae. Quantification of the area of immune cell infiltrate and frequency of observations, was significantly higher in the FIC cats in both the bladder and urethra LP (Figure 4; Kolmogorov-Smirnov test, *p* = 0.02).

**Figure 5:**
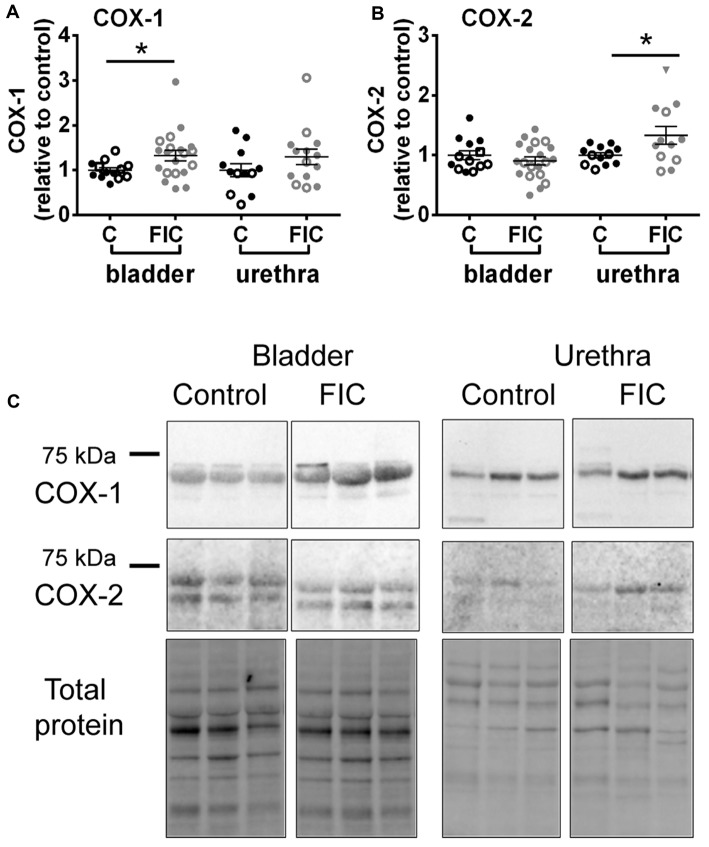
Cyclooxygenase (COX)-1 and COX-2 expression in the mucosa of the bladder and urethra. **(A)** COX-1 expression in the mucosa of the bladder (circles) and urethra (triangles) from control (*n* = 13 bladders and 12 urethral tissues) and FIC (*n* = 20 bladder and 14 urethral tissues) cats. Statistical significance was tested using Mann-Whitney test (bladder *p* = 0.03, urethra *p* = 0.31). **(B)** COX-2 expression in the mucosa of the bladder (circles) and urethra (triangles) from control (*n* = 13 bladders and 12 urethral tissues) and FIC (*n* = 20 bladder and 12 urethral tissues) cats. Statistical significance was tested using Mann-Whitney test (bladder *p* = 0.47, urethra *p* = 0.047). Data are presented as single points from individual cats with mean ± SEM. Asterisk (*) indicates *p* < 0.05. In all panels, each symbol represents data from one animal. Open circles represent males, closed circles females and open squares represent undocumented sex. **(C)** Representative blots from bladder and urethra indicating the expression of COX-1 and COX-2. Each of the three lines (control and FIC) represents tissue from a single cat probed for COX-1 (upper panel) COX-2 middle panel, and total protein (lower panel) which was used for normalization. The expected band sizes for COX-1 and COX-2 are 75 kDa.

To further investigate inflammatory pathways, we assessed the expression of constitutively expressed COX-1 enzyme and inducible isozyme, COX-2 in the mucosa of the bladder and urethra. COX-1 was significantly increased in the FIC bladder mucosa, and COX-2 was significantly increased in the FIC urethral mucosa, compared to control (Figure 5).

**Figure 6:**
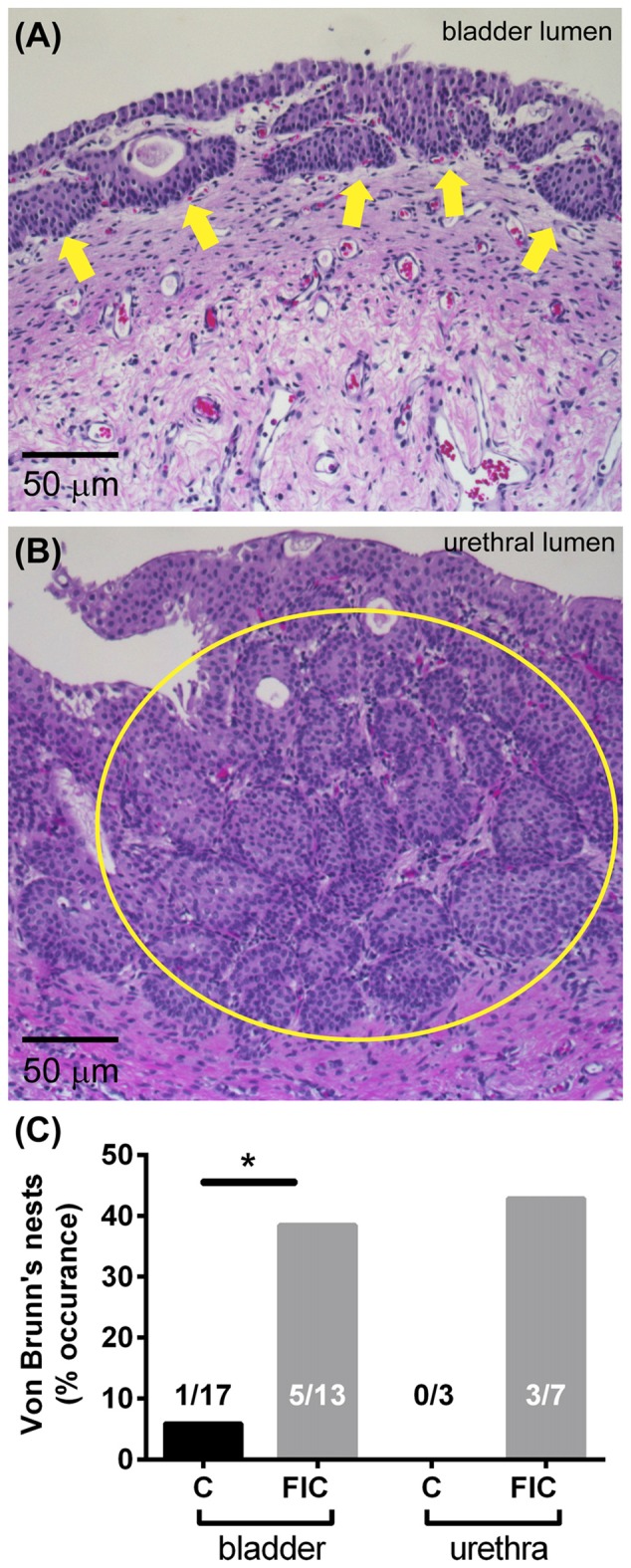
Von Brunn’s nests in the bladder and urethra.** (A)** Example of von Brunn’s nests indicated by yellow arrows, in the bladder of an FIC cat. **(B)** Example of von Brunn’s nests (inside the yellow circle) in the urethra of a FIC cat. **(C)** Percentage of control (black) and FIC (gray) cats presenting von Brunn’s nests in the bladder and urethra. Numbers on bar graphs represent the number of cats exhibiting von Brunn’s nests and the total number of cats assessed. Statistical significance was tested using Chi-square test: *p* = 0.02 for bladder and *p* = 0.17 for urethra. Asterisk (*) indicates *p* < 0.05.

**Figure 7:**
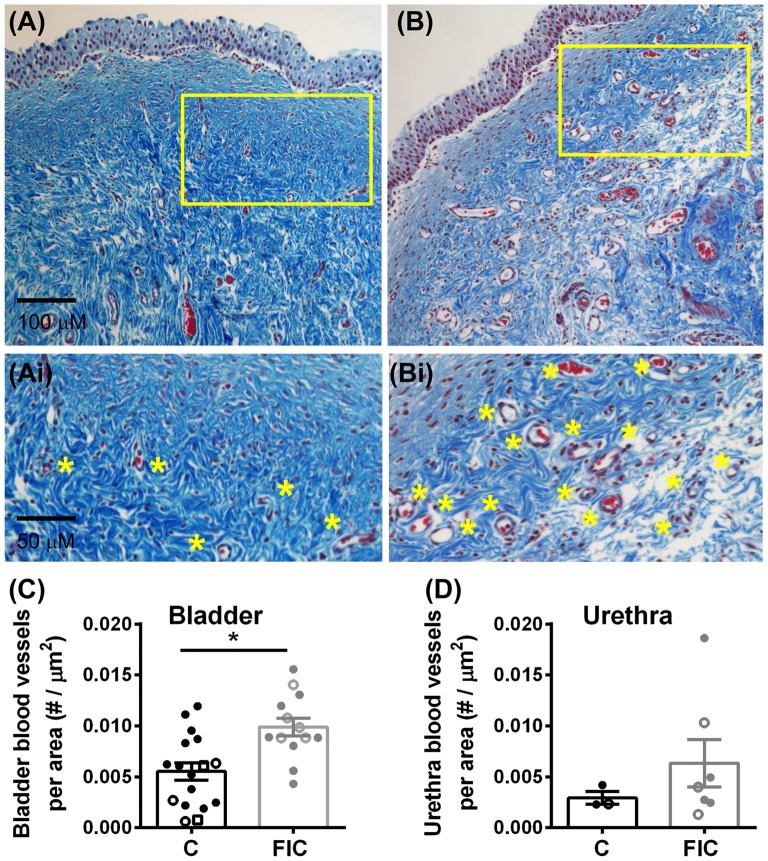
Increased angiogenesis in LP of the bladder and urethra. **(A,B)** Example of blood vessels in Mason trichrome stained tissues from control **(A)** and FIC **(B)** cat bladder. Insets **(Ai,Bi)** are magnified images of the areas outlined in yellow in **(A,B)**, to illustrate blood vessels (indicated by yellow stars placed next to the vessels). **(C,D)** Number of blood vessels per area of tissue in the bladder **(C)** and urethra **(D)** from control (black; *n* = 17 bladder and *n* = 3 urethral tissues; Mann-Whitney test *p* = 0.024) and FIC (gray; *n* = 13 bladder and *n* = 7 urethral tissues; Mann-Whitney test *p* = 0.3) cats. Each symbol represents data from one animal. Open circles represent males, closed circles females and open squares represent undocumented sex. Statistical significance was tested using Mann-Whitney test. Asterisk (*) indicates *p* < 0.05.

Chronic inflammation often leads to tissue remodeling. We evaluated tissue remodeling in FIC bladder and urethra by assessing the presence of von Brunn’s nests and changes in vascularization and elastin. Von Brunn’s nests are defined as groups of urothelial cells derived from the invagination of the surface urothelium into the LP (Young, 2009; e.g., in Figures 6A,B). These nests were present in both male and female bladder and urethral tissues. Quantification indicated their presence in 38% (5 out of 13; 2 of 8 females and 3 of 5 males) of bladder tissues and 43% (3 out of 7; 3 of 4 females and 0 of 3 males) of urethral tissues from FIC cats in and in 6% (1 out of 17; 1 of 12 females and 0 of 3 males; 2 unknown) of bladder tissues and 0% (0 out of 3; 1 male and 2 females) of urethral tissues from control cats (Figure 6C; Chi-square test *p* = 0.02 for bladder and *p* = 0.17 for urethra). Angiogenesis was assessed by counting the number of blood vessels in the LP. In the bladder this number was significantly higher in cats with FIC vs. control cats (Figures 7A–C). In the urethra, several FIC samples showed a marked increase in the number of blood vessels, however statistical significance was not reached likely due to the limited number of samples (Figure 7D). No significant correlation between age and number of blood vessels per area was observed in either control or FIC cats (Pearson correlation *r* was 0.16 and 0.02 for bladder and −0.19 and 0.25 for urethra from control and FIC cats, respectively).

**Figure 8:**
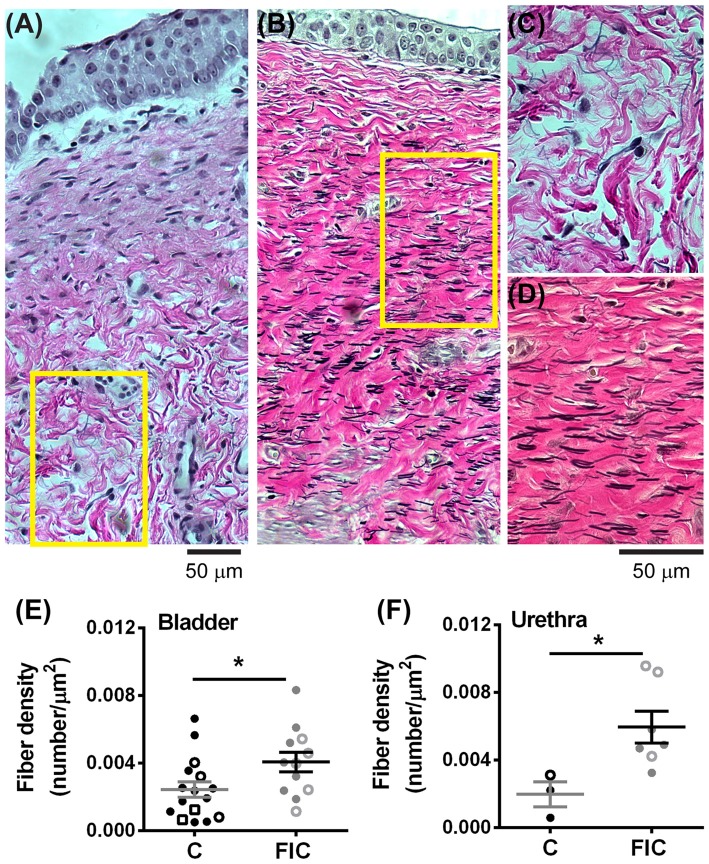
Increased elastin expression in the LP of the bladder and urethra. **(A,B)** Example of elastin fibers detected by Verhoeff-Van Gieson staining, in the bladder from control **(A)** and FIC **(B)** cats. **(C,D)** High magnification of the areas outlined in yellow in **(A,B)**. Note black fibers are more prominent in the FIC tissue **(B,D)**. **(E,F)** Elastin fiber density (quantified as number of fibers per tissue area) in the bladder **(E)** and urethra **(F)**. Data are from 16 bladders and three urethrae from control cats shown in black, and 12 bladders and seven urethrae from FIC cats shown in gray. Each symbol represents data from one animal. Open circles represent males, closed circles females and open squares represent undocumented sex. Statistical significance was evaluated using Mann-Whitney test. Asterisks (*) indicate *p* < 0.05 (*p* = 0.03 and *p* = 0.01 for bladder and urethra, respectively).

Elastin is a structural protein associated with inflammatory conditions and tissue remodeling. Elastin content in the LP was assessed by quantification of fiber density (Figure 8). This analysis showed statistically significant increases in fiber density in the LP tissue from FIC vs. control cats for both bladder and urethra (Figures 8E,F). No significant correlation between age and fiber density was observed in either control or FIC cats (Pearson correlation *r* was 0.08 and −0.24 for bladder and −0.15 and 0.33 for urethra from control and FIC cats, respectively). No difference in average fiber length was found between FIC and control (bladder control: 13.7 ± 1.0 μm, *n* = 16; bladder FIC: 12.3 ± 0.9 μm, *n* = 12; urethra control: 10.1 ± 2.3 μm, *n* = 3; urethra FIC: 11.2 ± 2.2 μm, *n* = 7; unpaired *t*-test > 0.05).

Together, these results suggest that an increase in inflammatory/pro-angiogenic factors within the bladder urothelium and LP may underlie tissue remodeling and neovascularization.

### Ultrastructural Investigation of the Urethral Epithelium Showed No Significant Change in FIC

**Figure 9:**
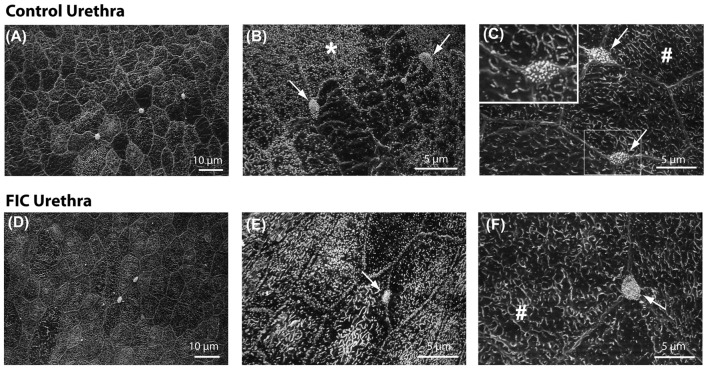
Urethral epithelial structure. **(A–F)** Scanning electron microscopy representative images of proximal urethral epithelium from control **(A–C)** and FIC **(D–F)** cats. **(A,D)** Low magnification views of representative areas. **(B,E)** High magnification areas from control **(B)** and FIC **(E)**, illustrating cells covered by short microvilli, indicated by *. **(C,F)** High magnification areas from control **(C)** and FIC **(F)** illustrating cells similar to bladder umbrella cells, indicated by ^#^. Arrows in **(B,C,E,F)**, and inset in **(C)** illustrate paraneurons, characterized by a tuft of microvilli-like appearance and located at the intersection of large polygonal cells (inset **C**).

We previously reported that FIC bladder urothelium exhibited morphological changes including patches of denuded urothelium (Lavelle et al., 2000). In the present study, we employed scanning electron microscopy (SEM) to determine if the epithelium lining the proximal urethra of FIC cats exhibited similar ultrastructural changes. In control animals (*n* = 3; one female, two males), this analysis revealed a mixed population of epithelial cells connected by junctional complexes (Figure 9). The first population was similar in appearance to bladder umbrella cells, displaying polygonal apices and covered with microplicae (# in Figures 9C,F). The second population also had a polygonal appearance, but their apical surfaces were covered with short microvilli (star in Figure 9B). These cells predominated in more caudal portions of the urethra. The third cell population were paraneurons (arrows in Figures 9B,C,E,F, and inset in Figure 9C), previously characterized as containing an apical tuft of microvilli (Dixon et al., 1973; Håkanson et al., 1974; Ramsdale, 1974). The apical boundary of paraneurons appeared smaller in size than adjacent epithelial cells, they were positioned at the intersection of the larger epithelial cells, and were not evident in the bladder urothelium. A similar analysis of the FIC proximal urethra (*n* = 3; two females, one male) did not identify any gross qualitative differences between the ultrastructure of control vs. FIC epithelium, or obvious differences in the number, appearance or distribution of paraneurons (Figures 9D–F).

## Discussion

This study investigated alterations in the tissue architecture and proteins associated with chronic inflammation and tissue remodeling in the bladder and proximal urethral mucosa obtained from cats diagnosed with FIC, a naturally occurring model of IC/BPS. The major findings include increased suburothelial proliferation, evidenced by mucosal von Brunn’s nests, alterations in the number of mast cells, elastin, accumulation of leukocytes, and neovascularization in the LP, all consistent with chronic inflammation and tissue remodeling. These alterations were present in both males and females. Although the sequence of events and biological responses are complex, these changes can lead to alterations in communication between epithelium, nerves, SM and vasculature and can result in urgency, frequency and pain in IC/BPS.

Two types of IC/BPS have been distinguished based on histological markers: Type I, non-ulcerative and Type II, ulcerative (also known as Hunner’s ulcers). Most patients (~90%), both human and feline, typically present with Type I form of IC/BPS. While Type II form appears to be an inflammatory disease intrinsic to the bladder, Type I form might be neuropathic in origin. In the present study, gross investigation of the bladders of FIC cats revealed no distinctive ulcerative areas of inflammation on the bladder wall. However, microscopic investigation indicated that a number of markers associated with inflammation were present and/or upregulated in the LP of the bladder and proximal urethra. Thus, it is possible that variable combinations of the presence of a subclinical infection, genetic susceptibility, aging, autoimmunity and sterile inflammation (Fleshner et al., 2017) may initiate a chronic inflammatory response that over time results in urothelial injury.

### Inflammatory Markers in Bladder and Urethra Mucosa and Implications for FIC

Previous studies indicated that inflammatory factors, such as leukotriene D4, antiproliferative factor (APF) and mast cells, are increased in the bladder of IC/BPS patients, and are often correlated with irritative voiding symptoms and pain (Lynes et al., 1990; Elbadawi and Light, 1996; Bouchelouche et al., 2001; Tomaszewski et al., 2001; Yoshimura and Chancellor, 2001; Keay et al., 2003, 2014). Our results in tissue from cats with FIC further extend these observations. Several histological findings, including an increased number of de-granulated mast cells (Figures 2, 3), increase in COX-1 and COX-2 expression (Figure 5) and accumulation of leukocytes (Figure 4), are consistent with chronic inflammation in the bladder and urethra mucosa. Although a non-specific marker of inflammation (Theoharides et al., 2015), mast cells have been regarded by urologists as the hallmark of IC/BPS, as biopsies of patients with ulcerative and non-ulcerative types of IC have shown increased numbers (Letourneau et al., 1992, 1996; Theoharides et al., 1995, 2001; Pang et al., 1996; Sant et al., 2007; Larsen et al., 2008; Kim et al., 2017). Mast cell numbers also are increased in some rodent models of cystitis, including rats exposed to immobilization stress (Spanos et al., 1997), water avoidance stress (Smith et al., 2011) and rodents with virus induced cystitis (Jasmin et al., 2000; Jasmin and Janni, 2003). Injury and/or alterations of the urothelial cells may result in production of cytokines and NGF (Liu and Kuo, 2007, 2012; Birder et al., 2010), which can stimulate proliferation and/or activation of mast cells (Sant et al., 2007). These in turn, release vasoactive and inflammatory mediators (e.g., histamine, kinins, cytokines, prostaglandins and nitric oxide), triggering inflammation and hyperexcitability of neuronal endings (Sant et al., 2007), which could increase the excitability of primary afferent neurons and contribute to bladder hypersensitivity and visceral pain.

Consistent with inflammatory processes, we found that COX-1 and COX-2 were increased in the mucosa of the bladder and urethra, respectively (Figure 5). COX-1 is expressed constitutively in most cells, including basal urothelial cells (Rahnama’i et al., 2010), and believed to be the main source of prostanoids that serve housekeeping functions (Dubois et al., 1998). The urothelial cells produce several prostaglandins including PGE2 (Jeremy et al., 1984; Pinna et al., 2000). Studies have shown that patients with ulcerative type II IC/BPS show increased urine levels of PGE2 (Wada et al., 2015), whereas in the general IC/BPS population urine levels of PGE2 may not differ from those of controls (Liu et al., 2010). Several types of PGE2 receptors are located on urothelial cells, afferent nerves and interstitial cells in the LP (Chuang et al., 2010). Their activation, as shown by instillation of PGE2 into the normal bladder, results in a hyperactive bladder, in part mediated by increased firing of Aδ and C-afferent nerves (Ritter et al., 2009; Aizawa et al., 2010; Kuga et al., 2016). Thus, a basal constitutive increase in PGE2, via upregulation of COX-1 in the bladder, may be activating afferent nerves and contributing to bladder hypersensitivity and pain in IC/BPS. COX-2 is an inducible enzyme, responsible for producing prostanoids, including PGE2, at the site of the inflammation. Although less is known about COX-2 expression in the urethra of IC/BPS patients, in conditions associated with urethra inflammation, such as urethra strictures as a result of transurethral resection of the prostate, treatment with COX-2 inhibitors improves voiding and prevents the development of urethra strictures (Sciarra et al., 2005), suggesting that stabilizing the levels of COX-2 is beneficial (at least in cases of acute iatrogenic inflammation). Upregulation of COX-2 in the proximal urethra of FIC cats may result in increased release of prostaglandins and subsequent activation of afferent nerves. Since this region of the urethra is densely innervated by sensory fibers (Barry et al., 2017; Kullmann et al., 2018) this may result in altered voiding sensations, urethral instability and pain (Kamo et al., 2004; Birder et al., 2014; Wyndaele et al., 2016).

Another interesting finding was infiltration of immune system cells, including neutrophils, in the LP of bladder and urethra of FIC cats (Figure 4). Although a number of studies in other systems suggest that neutrophils are major players in inflammatory responses, including the production of reactive oxidative species (ROS) and release of toxic factors (Yang et al., 2016), less is known about the role of neutrophils in the pathogenesis of IC/BPS. Increased infiltration of neutrophils in LP was reported in cat urethra (Kullmann et al., 2013) and rabbit bladder (Elgebaly et al., 1992) after acute acetic acid instillation into the bladder, and it was correlated with bladder instability and altered urethral function. Importantly, the urine of patients with IC/BPS contains higher levels of neutrophil chemotactic factors and urinary neutrophil elastase than the urine of healthy controls (Elgebaly et al., 1992; Kuromitsu et al., 2008). Moreover, urinary neutrophil elastase concentration was found to be higher in a subset of IC/BPS patients that had smaller bladder capacity and pain compared to other subgroups of IC patients with larger bladder capacity or healthy volunteers (Kuromitsu et al., 2008). Neutrophil chemotactic factors, including bacterial peptides, products of complement activation, extracellular matrix degradation products, platelet activating factors, and a number of cytokines, attract neutrophils at the site of injury and cause the release of toxic factors from neutrophils, one of which is neutrophil elastase. This enzyme is capable of degrading many extracellular matrix proteins, including elastin (Yang et al., 2016). In the lung this results in decreased compliance and stiffness of the tissue (Pandey et al., 2017). Thus, neutrophil elastase may play a similar role in the bladder and urethra of FIC and IC/BPS patients. Our results showing increased in elastin fibers in the bladder and urethral LP (Figure 8), may reflect a compensatory response to perpetual elastin degradation by this enzyme. Taken together, accumulation of immune system cells in the LP supports altered immune response and could contribute to tissue remodeling in IC/BPS.

### Tissue Remodeling and Potential Impact of Altered Matrix on Urothelial Function

Several histological findings suggest remodeling of the LP of both bladder and urethra in FIC. First, ~40% of bladders and ~ 50% of urethrae of FIC cats (both males and females) displayed von Brunn’s nests (Figure 6), which are groups of non-neoplastic reactive lesions found in the superficial LP that arise from invagination of the surface urothelium (Young, 2009; Harik and O’Toole, 2012). These nests stain for cell cycle inhibitors (p27) and markers for terminally differentiated cells (cytokeratin 20), and lack neoplastic markers such as proliferative markers (MIB-1) and cellular tumor markers (p53; Volmar et al., 2003). The nests are suggestive of tissue remodeling likely as a result of chronic irritation of the bladder mucosa (Harik and O’Toole, 2012) and have also been reported in human bladders with neurogenic detrusor overactivity (Brady et al., 2004). Another indication of tissue remodeling is an increase in number of blood vessels (Figure 7), thought to supply the extra nutrients needed during intense wound healing and remodeling. Although we did not investigate the expression of the vascular endothelial growth factor (VEGF), which plays a significant role in remodeling of blood vessels, previous studies reported changes in VEGF and/or VEGF receptors and signaling pathways in the bladder of patients with IC/BPS (Tamaki et al., 2004; Saban R. et al., 2008; Kiuchi et al., 2009), and rodent models of bladder inflammation (Saban M. R. et al., 2008; Saban R. et al., 2008). Consistent with tissue remodeling, other studies reported that fibronectin and galectin-7, proteins that play important roles in cell adhesion, migration, differentiation and wound healing, were significantly upregulated in the urine of cats with FIC (Lemberger et al., 2011; Treutlein et al., 2012, 2013).

The composition and structure of the extracellular matrix is crucial to cell phenotype, repair, proliferation, differentiation, survival and importantly, function. Recent studies using bioaxial stretch of urinary bladders in combination with imaging methods have revealed a layer-dependent role of collagen fiber recruitment during bladder stretch (Cheng et al., 2018). Alterations in the LP suggest that recruitment of extracellular matrix fibers during bladder distention can impact on epithelial release of transmitters. Indeed, previous studies have shown a number of functional changes in these cells, including abnormal release of ATP (Birder et al., 2003), platelet-activating factor (PAF; Kispert et al., 2017), aberrant differentiation associated with increased production of the APF (Keay et al., 2014), increased purinergic, TRPV1, and muscarinic signaling (Keay et al., 2014), all contributing to the pathophysiology of the disease.

### Urethral Epithelium and Its Role in FIC

The urethral epithelium is a stratified epithelium composed of several cell layers forming an interface between the lumen and the underlying neuronal, vasculature, connective, and muscular tissues. Von Brunn’s nests in the urethra were found in the epithelium in ~40% of cats with FIC (Figure 4). However, the integrity of the luminal surface of this epithelium did not appear to be altered in tissue from cats with FIC (Figure 9). This is in contrast to previous observations of the bladder epithelium of cats with FIC and humans with IC/BPS, where denuded epithelium and apoptosis have been reported (Shie et al., 2012). These differences may be related to the underlying function of bladder vs. urethral epithelium; the former may serve as a barrier, while the latter may have more of a sensory role. Although how sensory information from the urethral lumen is sensed and transmitted to the afferent nerves is not understood, one possibility is via paraneuron-mediated signaling mechanisms. Paraneurons are specialized cells within the urethra that express transmitters including ACh (Deckmann et al., 2014), 5-HT (Håkanson et al., 1974; Vittoria et al., 1990; Kullmann et al., 2018) and somatostatin (Vittoria et al., 1990), and may release these transmitters in response to mechanical and chemical stimuli. Microvilli-expressing cells (Figure 9), which are not present in the bladder epithelium (Apodaca/Ruiz unpublished observations), were located within the proximal urethra and appeared unchanged in number or morphology in the tissue from cats with FIC. In rodents, cells with similar morphology are located in the proximity of afferent nerve fibers, including C-fibers, and may serve as chemo-detectors for bacteria and harmful substances entering the urinary tract and/or may be involved in nociception (Deckmann et al., 2014; Kullmann et al., 2018). Thus it is possible that the microvilli- expressing paraneurons in cat urethra could also serve similar functions. Although no structural changes in the epithelium and paraneurons in FIC cats were found, it remains to be investigated whether functional changes in paraneuron properties that impact sensory processing may occur.

## Conclusion

Several hypotheses have been proposed for factors contributing to the etiology of IC/BPS, including epithelial dysfunction, neurogenic inflammation, mast cell upregulation, autoimmunity and infection, growth factors (e.g., NGF, VEGF, PD-EFGF) and genetics (Yoshimura and Chancellor, 2001). Our results in cats with FIC provide evidence for an important role of inflammatory pathways and tissue remodeling as a result of chronic inflammation in IC/BPS. Though the variability observed in several parameters suggests additional contributing factors, such as age which was significantly higher in FIC cats compared to controls, this study has provided new evidence that FIC is accompanied by remodeling of the LP of the bladder and proximal urethra, which is present in both males and females. This may alter the communication of sensory information between different components (epithelium, nerves, muscle) of the bladder and urethra. Thus, disruptions in the mucosal signaling pathway within the bladder and proximal urethra may contribute to altered sensations and pain that occur in FIC as well as in IC/BPS patients. Our results, together with previous findings indicate an important role for chronic inflammation, tissue remodeling, and fibrosis in the pathogenesis of IC/BPS.

Our results also fit with clinical experience treating cats with the syndrome. Reduction of perception of threat by environmental modification has been found to resolve both bladder signs and sickness behaviors in both laboratory (Stella et al., 2011) and clinical studies (Buffington et al., 2006). These results suggest that the inflammatory changes seen may result from centrally-mediated sterile inflammation (Fleshner et al., 2017). Further reinforcing this explanation are the similarities with bladder changes found in rodents subjected to water avoidance stress (Smith et al., 2011; Lee et al., 2015; Ackerman et al., 2016; Greenwood-Van Meerveld and Johnson, 2017; Matos et al., 2017; Wang et al., 2017), suggesting that IC/BPS may be a syndrome affecting the bladder rather than a bladder-based syndrome.

## Author Contributions

FAK and LAB: designed the study and wrote the manuscript. FAK, BMM, AML, DG, SEG, ASW-J, WGR, IVZ, YI, JRR, SIB and GA: designed, collected and analyzed data. CATB: diagnosed and characterized the cats, and provided them for this study. AJK and LAB: supervised personnel.

## Conflict of Interest Statement

The authors declare that the research was conducted in the absence of any commercial or financial relationships that could be construed as a potential conflict of interest.

## References

[B1] Ackerman A. L. Jellison F. C. Lee U. J. Bradesi S. Rodríguez L. V. (2016). The Glt1 glutamate receptor mediates the establishment and perpetuation of chronic visceral pain in an animal model of stress-induced bladder hyperalgesia. Am. J. Physiol. Renal Physiol. 310, F628–F636. 10.1152/ajprenal.00297.2015 26697981 PMC5504462

[B2] Aizawa N. Igawa Y. Nishizawa O. Wyndaele J. J. (2010). Effects of CL316,243, a β 3-adrenoceptor agonist and intravesical prostaglandin E2 on the primary bladder afferent activity of the rat. Neurourol. Urodyn. 29, 771–776. 10.1002/nau.20826 19816919

[B3] Barry C. M. Ji E. Sharma H. Yap P. Spencer N. J. Matusica D.. (2017). Peptidergic nerve fibers in the urethra: morphological and neurochemical characteristics in female mice of reproductive age. Neurourol. Urodyn. [Epub ahead of print]. 10.1002/nau.23434 29053899

[B4] Birder L. A. Barrick S. R. Roppolo J. R. Kanai A. J. de Groat W. C. Kiss S.. (2003). Feline interstitial cystitis results in mechanical hypersensitivity and altered ATP release from bladder urothelium. Am. J. Physiol. Renal Physiol. 285, F423–F429. 10.1152/ajprenal.00056.2003 12759226

[B5] Birder L. A. de Wachter S. Gillespie J. Wyndaele J. J. (2014). Urethral sensation: basic mechanisms and clinical expressions. Int. J. Urol. 21, 13–16. 10.1111/iju.12349 24807486

[B6] Birder L. A. Ruan H. Z. Chopra B. Xiang Z. Barrick S. Buffington C. A.. (2004). Alterations in P2X and P2Y purinergic receptor expression in urinary bladder from normal cats and cats with interstitial cystitis. Am. J. Physiol. Renal Physiol. 287, F1084–F1091. 10.1152/ajprenal.00118.2004 15251862

[B7] Birder L. A. Wolf-Johnston A. Buffington C. A. Roppolo J. R. de Groat W. C. Kanai A. J. (2005). Altered inducible nitric oxide synthase expression and nitric oxide production in the bladder of cats with feline interstitial cystitis. J. Urol. 173, 625–629. 10.1097/01.ju.0000145900.22849.1d 15643277

[B8] Birder L. A. Wolf-Johnston A. S. Chib M. K. Buffington C. A. Roppolo J. R. Hanna-Mitchell A. T. (2010). Beyond neurons: involvement of urothelial and glial cells in bladder function. Neurourol. Urodyn. 29, 88–96. 10.1002/nau.20747 20025015 PMC2910110

[B9] Bouchelouche K. Nordling J. Hald T. Bouchelouche P. (2001). Treatment of interstitial cystitis with montelukast, a leukotriene D_4_ receptor antagonist. Urology 57:118. 10.1016/s0090-4295(01)01066-4 11378098

[B10] Brady C. M. Apostolidis A. N. Harper M. Yiangou Y. Beckett A. Jacques T. S.. (2004). Parallel changes in bladder suburothelial vanilloid receptor TRPV1 and pan-neuronal marker PGP9.5 immunoreactivity in patients with neurogenic detrusor overactivity after intravesical resiniferatoxin treatment. BJU Int. 93, 770–776. 10.1111/j.1464-410x.2003.04722.x 15049988

[B13] Buffington C. A. T. (2004). Comorbidity of interstitial cystitis with other unexplained clinical conditions. J. Urol. 172, 1242–1248. 10.1097/01.ju.0000137953.49304.6c 15371816

[B11] Buffington C. A. T. (2011). Idiopathic cystitis in domestic cats—beyond the lower urinary tract. J. Vet. Intern. Med. 25, 784–796. 10.1111/j.1939-1676.2011.0732.x 21564297 PMC3760680

[B12] Buffington C. A. T. Chew D. J. Woodworth B. E. (1999). Feline interstitial cystitis. J. Am. Vet. Med. Assoc. 215, 682–687. 10476717

[B14] Buffington C. A. T. Westropp J. L. Chew D. J. Bolus R. R. (2006). Clinical evaluation of multimodal environmental modification in the management of cats with lower urinary tract signs. J. Feline Med. Surg. 8, 261–268. 10.1016/j.jfms.2006.02.002 16616567 PMC10822542

[B15] Cheng F. Birder L. A. Kullmann F. A. Hornsby J. Watton P. N. Watkins S.. (2018). Layer-dependent role of collagen recruitment during loading of the rat bladder wall. Biomech. Model. Mechanobiol. 17, 403–417. 10.1007/s10237-017-0968-5 29039043 PMC5845476

[B16] Chuang Y. C. Yoshimura N. Huang C. C. Wu M. Tyagi P. Chancellor M. B. (2010). Expression of E-series prostaglandin (EP) receptors and urodynamic effects of an EP4 receptor antagonist on cyclophosphamide-induced overactive bladder in rats. BJU Int. 106, 1782–1787. 10.1111/j.1464-410X.2010.09260.x 20346049 PMC3102303

[B17] Danziger Z. C. Grill W. M. (2016). Sensory and circuit mechanisms mediating lower urinary tract reflexes. Auton. Neurosci. 200, 21–28. 10.1016/j.autneu.2015.06.004 26119358 PMC4670817

[B18] Deckmann K. Filipski K. Krasteva-Christ G. Fronius M. Althaus M. Rafiq A.. (2014). Bitter triggers acetylcholine release from polymodal urethral chemosensory cells and bladder reflexes. Proc. Natl. Acad. Sci. U S A 111, 8287–8292. 10.1073/pnas.1402436111 24843119 PMC4050540

[B19] Dixon J. S. Gosling J. A. Ramsdale D. R. (1973). Urethral chromaffin cells. A light and electron microscopic study. Z. Zellforsch. Mikrosk. Anat. 138, 397–406. 10.1007/BF00307101 4735903

[B20] Dubois R. N. Abramson S. B. Crofford L. Gupta R. A. Simon L. S. Van De Putte L. B.. (1998). Cyclooxygenase in biology and disease. FASEB J. 12, 1063–1073. 10.1096/fasebj.12.12.1063 9737710

[B21] Elbadawi A. E. Light J. K. (1996). Distinctive ultrastructural pathology of nonulcerative interstitial cystitis: new observations and their potential significance in pathogenesis. Urol. Int. 56, 137–162. 10.1159/000282832 8860736

[B22] Elgebaly S. A. Allam M. E. Walzak M. P. Jr. Oselinsky D. Gillies C. Yamase H. (1992). Urinary neutrophil chemotactic factors in interstitial cystitis patients and a rabbit model of bladder inflammation. J. Urol. 147, 1382–1387. 10.1016/s0022-5347(17)37578-x 1569692

[B23] Fleshner M. Frank M. Maier S. F. (2017). Danger signals and inflammasomes: stress-evoked sterile inflammation in mood disorders. Neuropsychopharmacology 42, 36–45. 10.1038/npp.2016.125 27412959 PMC5143484

[B24] Gamper M. Viereck V. Eberhard J. Binder J. Moll C. Welter J.. (2013). Local immune response in bladder pain syndrome/interstitial cystitis ESSIC type 3C. Int. Urogynecol. J. 24, 2049–2057. 10.1007/s00192-013-2112-0 23670165 PMC3838592

[B25] Gevaert T. De Vos R. Everaerts W. Libbrecht L. Van Der Aa F. van den Oord J.. (2011). Characterization of upper lamina propria interstitial cells in bladders from patients with neurogenic detrusor overactivity and bladder pain syndrome. J. Cell. Mol. Med. 15, 2586–2593. 10.1111/j.1582-4934.2011.01262.x 21251216 PMC4373427

[B26] Greenwood-Van Meerveld B. Johnson A. C. (2017). Stress-induced chronic visceral pain of gastrointestinal origin. Front. Syst. Neurosci. 11:86. 10.3389/fnsys.2017.00086 29213232 PMC5702626

[B27] Håkanson R. Larsson L. I. Sjöberg N. O. Sundler F. (1974). Amine-producing endocrine-like cells in the epithelium of urethra and prostate of the guinea-pig. A chemical, fluorescence histochemical, and electron microscopic study. Histochemie 38, 259–270. 10.1007/bf00493124 4134747

[B28] Hanno P. Nordling J. Fall M. (2011). Bladder pain syndrome. Med. Clin. North Am. 95, 55–73. 10.1016/j.mcna.2010.08.014 21095411

[B29] Harik L. R. O’Toole K. M. (2012). Nonneoplastic lesions of the prostate and bladder. Arch. Pathol. Lab. Med. 136, 721–734. 10.5858/arpa.2011-0584-RA 22742546

[B30] Hauser P. J. Dozmorov M. G. Bane B. L. Slobodov G. Culkin D. J. Hurst R. E. (2008). Abnormal expression of differentiation related proteins and proteoglycan core proteins in the urothelium of patients with interstitial cystitis. J. Urol. 179, 764–769. 10.1016/j.juro.2007.09.022 18082196 PMC2652890

[B31] Hauser P. J. VanGordon S. B. Seavey J. Sofinowski T. M. Ramadan M. Abdullah S.. (2015). Abnormalities in expression of structural, barrier and differentiation related proteins, and chondroitin sulfate in the urothelium of cats with feline interstitial cystitis mimic those seen in human interstitial cystitis. J. Urol. 194, 571–577. 10.1016/j.juro.2015.01.090 25636658 PMC4699667

[B32] Jasmin L. Janni G. (2003). Experimental neurogenic cystitis. Adv. Exp. Med. Biol. 539, 319–335. 10.1007/978-1-4419-8889-8_24 15088915

[B33] Jasmin L. Janni G. Ohara P. T. Rabkin S. D. (2000). CNS induced neurogenic cystitis is associated with bladder mast cell degranulation in the rat. J. Urol. 164, 852–855. 10.1097/00005392-200009010-00061 10953167

[B34] Jeremy J. Y. Mikhailidis D. P. Dandona P. (1984). The rat urinary bladder produces prostacyclin as well as other prostaglandins. Prostaglandins Leukot. Med. 16, 235–248. 10.1016/0262-1746(84)90074-X 6441939

[B35] Jung S. Y. Fraser M. O. Ozawa H. Yokoyama O. Yoshiyama M. De Groat W. C.. (1999). Urethral afferent nerve activity affects the micturition reflex; implication for the relationship between stress incontinence and detrusor instability. J. Urol. 162, 204–212. 10.1097/00005392-199907000-00069 10379788

[B36] Kamo I. Cannon T. W. Conway D. A. Torimoto K. Chancellor M. B. de Groat W. C.. (2004). The role of bladder-to-urethral reflexes in urinary continence mechanisms in rats. Am. J. Physiol. Renal Physiol. 287, F434–F441. 10.1152/ajprenal.00038.2004 15113743

[B38] Keay S. K. Birder L. A. Chai T. C. (2014). Evidence for bladder urothelial pathophysiology in functional bladder disorders. Biomed. Res. Int. 2014:865463. 10.1155/2014/865463 24900993 PMC4034482

[B37] Keay S. Zhang C. O. Shoenfelt J. L. Chai T. C. (2003). Decreased *in vitro* proliferation of bladder epithelial cells from patients with interstitial cystitis. Urology 61, 1278–1284. 10.1016/s0090-4295(03)00005-0 12809929

[B39] Kim A. Han J. Y. Ryu C. M. Yu H. Y. Lee S. Kim Y.. (2017). Histopathological characteristics of interstitial cystitis/bladder pain syndrome without Hunner lesion. Histopathology 71, 415–424. 10.1111/his.13235 28394416

[B40] Kispert S. E. Marentette J. Campian E. C. Isbell T. S. Kuenzel H. McHowat J. (2017). Cigarette smoke-induced urothelial cell damage: potential role of platelet-activating factor. Physiol. Rep. 5:e13177. 10.14814/phy2.13177 28270596 PMC5350181

[B41] Kiuchi H. Tsujimura A. Takao T. Yamamoto K. Nakayama J. Miyagawa Y.. (2009). Increased vascular endothelial growth factor expression in patients with bladder pain syndrome/interstitial cystitis: its association with pain severity and glomerulations. BJU Int. 104, 826–831; discussion 831. 10.1111/j.1464-410x.2009.08467.x 19298410

[B42] Kuga N. Tanioka A. Hagihara K. Kawai T. (2016). Modulation of afferent nerve activity by prostaglandin E2 upon urinary bladder distension in rats. Exp. Physiol. 101, 577–587. 10.1113/EP085418 26841236

[B43] Kullmann F. A. Chang H. H. Gauthier C. McDonnell B. M. Yeh J. C. Clayton D. R.. (2018). Serotonergic paraneurons in the female mouse urethral epithelium and their potential role in peripheral sensory information processing. Acta Physiol. 222:e12919. 10.1111/apha.12919 28719042 PMC5963688

[B44] Kullmann F. A. Wells G. I. Langdale C. L. Zheng J. Thor K. B. (2013). Stability of the acetic acid-induced bladder irritation model in α chloralose-anesthetized female cats. PLoS One 8:e73771. 10.1371/journal.pone.0073771 24040064 PMC3767621

[B45] Kumar V. Chapple C. R. Surprenant A. M. Chess-Williams R. (2007). Enhanced adenosine triphosphate release from the urothelium of patients with painful bladder syndrome: a possible pathophysiological explanation. J. Urol. 178, 1533–1536. 10.1016/j.juro.2007.05.116 17707056

[B46] Kuromitsu S. Yokota H. Hiramoto M. Morita S. Mita H. Yamada T. (2008). Increased concentration of neutrophil elastase in urine from patients with interstitial cystitis. Scand. J. Urol. Nephrol. 42, 455–461. 10.1080/00365590802025881 18609268

[B47] Larsen M. S. Mortensen S. Nordling J. Horn T. (2008). Quantifying mast cells in bladder pain syndrome by immunohistochemical analysis. BJU Int. 102, 204–207; discussion 207. 10.1111/j.1464-410x.2008.07576.x 18384636

[B48] Lavelle J. P. Meyers S. A. Ruiz W. G. Buffington C. A. Zeidel M. L. Apodaca G. (2000). Urothelial pathophysiological changes in feline interstitial cystitis: a human model. Am. J. Physiol. Renal Physiol. 278, F540–F553. 10.1152/ajprenal.2000.278.4.f540 10751214

[B49] Lee U. J. Ackerman A. L. Wu A. Zhang R. Leung J. Bradesi S.. (2015). Chronic psychological stress in high-anxiety rats induces sustained bladder hyperalgesia. Physiol. Behav. 139, 541–548. 10.1016/j.physbeh.2014.11.045 25449389

[B50] Leiby B. E. Landis J. R. Propert K. J. Tomaszewski J. E. Interstitial Cystitis Data Base Study Group. (2007). Discovery of morphological subgroups that correlate with severity of symptoms in interstitial cystitis: a proposed biopsy classification system. J. Urol. 177, 142–148. 10.1016/j.juro.2006.08.096 17162024

[B51] Lemberger S. I. Deeg C. A. Hauck S. M. Amann B. Hirmer S. Hartmann K.. (2011). Comparison of urine protein profiles in cats without urinary tract disease and cats with idiopathic cystitis, bacterial urinary tract infection, or urolithiasis. Am. J. Vet. Res. 72, 1407–1415. 10.2460/ajvr.72.10.1407 21962285

[B52] Letourneau R. Pang X. Sant G. R. Theoharides T. C. (1996). Intragranular activation of bladder mast cells and their association with nerve processes in interstitial cystitis. Br. J. Urol. 77, 41–54. 10.1046/j.1464-410x.1996.08178.x 8653316

[B53] Letourneau R. Sant G. R. el-Mansoury M. Theoharides T. C. (1992). Activation of bladder mast cells in interstitial cystitis. Int. J. Tissue React. 14, 307–312. 1306530

[B54] Liu H. T. Kuo H. C. (2007). Intravesical botulinum toxin A injections plus hydrodistension can reduce nerve growth factor production and control bladder pain in interstitial cystitis. Urology 70, 463–468. 10.1016/j.urology.2007.04.038 17905097

[B55] Liu H. T. Kuo H. C. (2012). Increased urine and serum nerve growth factor levels in interstitial cystitis suggest chronic inflammation is involved in the pathogenesis of disease. PLoS One 7:e44687. 10.1371/journal.pone.0044687 23028581 PMC3444462

[B56] Liu H. T. Tyagi P. Chancellor M. B. Kuo H. C. (2010). Urinary nerve growth factor but not prostaglandin E2 increases in patients with interstitial cystitis/bladder pain syndrome and detrusor overactivity. BJU Int. 106, 1681–1685. 10.1111/j.1464-410x.2009.08851.x 19751258

[B57] Lynes W. L. Flynn S. D. Shortliffe L. D. Stamey T. A. (1990). The histology of interstitial cystitis. Am. J. Surg. Pathol. 14, 969–976. 10.1097/00000478-199010000-00011 2403198

[B58] Matos R. Serrão P. Rodriguez L. Birder L. A. Cruz F. Charrua A. (2017). The water avoidance stress induces bladder pain due to a prolonged α1A adrenoceptor stimulation. Naunyn Schmiedebergs Arch. Pharmacol. 2017, 839–844. 10.1007/s00210-017-1384-1 28569366

[B59] Pandey K. C. De S. Mishra P. K. (2017). Role of proteases in chronic obstructive pulmonary disease. Front. Pharmacol. 8:512. 10.3389/fphar.2017.00512 28848433 PMC5550664

[B60] Pang X. Boucher W. Triadafilopoulos G. Sant G. R. Theoharides T. C. (1996). Mast cell and substance P-positive nerve involvement in a patient with both irritable bowel syndrome and interstitial cystitis. Urology 47, 436–438. 10.1016/s0090-4295(99)80469-5 8633418

[B61] Payne C. K. (2017). The State of BPS/IC: The Emperor Has No Clothes (AUA 2017). Available online at: https://www.urotoday.com/video-lectures/pelvic-health/video/mediaitem/788-embedded-media2017-07-05-16-32-47.html?utm_source=newsletter_4812&utm_medium=email&utm_campaign=september-is-ic-awareness-month-featuring-video-with-christopher-payne&acm=_4812

[B62] Pinna C. Zanardo R. Puglisi L. (2000). Prostaglandin-release impairment in the bladder epithelium of streptozotocin-induced diabetic rats. Eur. J. Pharmacol. 388, 267–273. 10.1016/s0014-2999(99)00833-x 10675736

[B63] Rahnama’i M. S. van Koeveringe G. A. Essers P. B. de Wachter S. G. de Vente J. van Kerrebroeck P. E.. (2010). Prostaglandin receptor EP1 and EP2 site in guinea pig bladder urothelium and lamina propria. J. Urol. 183, 1241–1247. 10.1016/j.juro.2009.11.004 20096878

[B64] Ramsdale D. R. (1974). Further observations on urethral chromaffin cells: an electron microscopic study. Cell Tissue Res. 148, 499–504. 10.1007/bf00221934 4365460

[B65] Ritter A. M. Martin W. J. Thorneloe K. S. (2009). The voltage-gated sodium channel Nav1.9 is required for inflammation-based urinary bladder dysfunction. Neurosci. Lett. 452, 28–32. 10.1016/j.neulet.2008.12.051 19146922

[B66] Robain G. Combrisson H. Mazieres L. (2001). Bladder response to urethral flow in the awake ewe. Neurourol. Urodyn. 20, 641–649. 10.1002/nau.1014 11574939

[B67] Roppolo J. R. Tai C. Booth A. M. Buffington C. A. de Groat W. C. Birder L. A. (2005). Bladder Aδ afferent nerve activity in normal cats and cats with feline interstitial cystitis. J. Urol. 173, 1011–1015. 10.1097/01.ju.0000145591.35569.9e 15711367

[B68] Saban M. R. Backer J. M. Backer M. V. Maier J. Fowler B. Davis C. A.. (2008). VEGF receptors and neuropilins are expressed in the urothelial and neuronal cells in normal mouse urinary bladder and are upregulated in inflammation. Am. J. Physiol. Renal Physiol. 295, F60–F72. 10.1152/ajprenal.00618.2007 18463314 PMC2494518

[B69] Saban R. Saban M. R. Maier J. Fowler B. Tengowski M. Davis C. A.. (2008). Urothelial expression of neuropilins and VEGF receptors in control and interstitial cystitis patients. Am. J. Physiol. Renal Physiol. 295, F1613–F1623. 10.1152/ajprenal.90344.2008 18815217 PMC2604836

[B70] Sant G. R. Kempuraj D. Marchand J. E. Theoharides T. C. (2007). The mast cell in interstitial cystitis: role in pathophysiology and pathogenesis. Urology 69, 34–40. 10.1016/j.urology.2006.08.1109 17462477

[B71] Sciarra A. Salciccia S. Albanesi L. Cardi A. D’Eramo G. Di Silverio F. (2005). Use of cyclooxygenase-2 inhibitor for prevention of urethral strictures secondary to transurethral resection of the prostate. Urology 66, 1218–1222. 10.1016/j.urology.2005.06.090 16360446

[B72] Shafik A. el-Sibai O. Ahmed I. (2003a). Effect of urethral dilation on vesical motor activity: identification of the urethrovesical reflex and its role in voiding. J. Urol. 169, 1017–1019. 10.1097/01.ju.0000046384.71563.51 12576835

[B73] Shafik A. Shafik A. A. El-Sibai O. Ahmed I. (2003b). Role of positive urethrovesical feedback in vesical evacuation. The concept of a second micturition reflex: the urethrovesical reflex. World J. Urol. 21, 167–170. 10.1007/s00345-003-0340-5 12898170

[B74] Shie J. H. Liu H. T. Kuo H. C. (2012). Increased cell apoptosis of urothelium mediated by inflammation in interstitial cystitis/painful bladder syndrome. Urology 79, 484.e7–484.e13. 10.1016/j.urology.2011.09.049 22310775

[B75] Slobodov G. Feloney M. Gran C. Kyker K. D. Hurst R. E. Culkin D. J. (2004). Abnormal expression of molecular markers for bladder impermeability and differentiation in the urothelium of patients with interstitial cystitis. J. Urol. 171, 1554–1558. 10.1097/01.ju.0000118938.09119.a5 15017219

[B76] Smith A. L. Leung J. Kun S. Zhang R. Karagiannides I. Raz S.. (2011). The effects of acute and chronic psychological stress on bladder function in a rodent model. Urology 78, 967.e1–967.e7. 10.1016/j.urology.2011.06.041 21868072 PMC3190050

[B77] Spanos C. Pang X. Ligris K. Letourneau R. Alferes L. Alexacos N.. (1997). Stress-induced bladder mast cell activation: implications for interstitial cystitis. J. Urol. 157, 669–672. 10.1016/s0022-5347(01)65247-9 8996395

[B78] Stella J. L. Lord L. K. Buffington C. A. (2011). Sickness behaviors in response to unusual external events in healthy cats and cats with feline interstitial cystitis. J. Am. Vet. Med. Assoc. 238, 67–73. 10.2460/javma.238.1.67 21194324 PMC3852887

[B79] Sun Y. Chai T. C. (2004). Up-regulation of P2X3 receptor during stretch of bladder urothelial cells from patients with interstitial cystitis. J. Urol. 171, 448–452. 10.1097/01.ju.0000099660.46774.3c 14665953

[B80] Tamaki M. Saito R. Ogawa O. Yoshimura N. Ueda T. (2004). Possible mechanisms inducing glomerulations in interstitial cystitis: relationship between endoscopic findings and expression of angiogenic growth factors. J. Urol. 172, 945–948. 10.1097/01.ju.0000135009.55905.cb 15311005

[B81] Theoharides T. C. Kempuraj D. Sant G. R. (2001). Mast cell involvement in interstitial cystitis: a review of human and experimental evidence. Urology 57, 47–55. 10.1016/s0090-4295(01)01129-3 11378050

[B82] Theoharides T. C. Sant G. R. el-Mansoury M. Letourneau R. Ucci A. A. Jr. Meares E. M. Jr. (1995). Activation of bladder mast cells in interstitial cystitis: a light and electron microscopic study. J. Urol. 153, 629–636. 10.1016/s0022-5347(01)67669-9 7861501

[B83] Theoharides T. C. Valent P. Akin C. (2015). Mast cells, mastocytosis, and related disorders. N. Engl. J. Med. 373, 163–172. 10.1056/NEJMra1409760 26154789

[B84] Tomaszewski J. E. Landis J. R. Russack V. Williams T. M. Wang L. P. Hardy C.. (2001). Biopsy features are associated with primary symptoms in interstitial cystitis: results from the interstitial cystitis database study. Urology 57, 67–81. 10.1016/s0090-4295(01)01166-9 11378053

[B85] Treutlein G. Deeg C. A. Hauck S. M. Amann B. Hartmann K. Dorsch R. (2013). Follow-up protein profiles in urine samples during the course of obstructive feline idiopathic cystitis. Vet. J. 198, 625–630. 10.1016/j.tvjl.2013.09.015 24257070

[B86] Treutlein G. Dorsch R. Euler K. N. Hauck S. M. Amann B. Hartmann K.. (2012). Novel potential interacting partners of fibronectin in spontaneous animal model of interstitial cystitis. PLoS One 7:e51391. 10.1371/journal.pone.0051391 23236492 PMC3517491

[B87] Vittoria A. La Mura E. Cocca T. Cecio A. (1990). Serotonin-, somatostatin- and chromogranin A-containing cells of the urethro-prostatic complex in the sheep. An immunocytochemical and immunofluorescent study. J. Anat. 171, 169–178. 1981998 PMC1257138

[B88] Volmar K. E. Chan T. Y. De Marzo A. M. Epstein J. I. (2003). Florid von Brunn nests mimicking urothelial carcinoma: a morphologic and immunohistochemical comparison to the nested variant of urothelial carcinoma. Am. J. Surg. Pathol. 27, 1243–1252. 10.1097/00000478-200309000-00008 12960809

[B89] Wada N. Ameda K. Furuno T. Okada H. Date I. Kakizaki H. (2015). Evaluation of prostaglandin E2 and E-series prostaglandin receptor in patients with interstitial cystitis. J. Urol. 193, 1987–1993. 10.1016/j.juro.2015.01.010 25595860

[B90] Wang Z. Chang H. H. Gao Y. Zhang R. Guo Y. Holschneider D. P.. (2017). Effects of water avoidance stress on peripheral and central responses during bladder filling in the rat: a multidisciplinary approach to the study of urologic chronic pelvic pain syndrome (MAPP) research network study. PLoS One 12:e0182976. 10.1371/journal.pone.0182976 28886046 PMC5590813

[B91] Wyndaele J.-J. Van de Borne S. Wyndaele M. De Wachter S. (2016). Sensation in the urethra during voiding studied in young healthy men. Bladder 3:e23 10.14440/bladder.2016.82

[B92] Yang H. Biermann M. H. Brauner J. M. Liu Y. Zhao Y. Herrmann M. (2016). New insights into neutrophil extracellular traps: mechanisms of formation and role in inflammation. Front. Immunol. 7:302. 10.3389/fimmu.2016.00302 27570525 PMC4981595

[B93] Yoshimura N. Chancellor M. B. (2001). Interstitial cystitis and bladder research: progress and future directions: highlights of the national institute of diabetes and digestive and kidney diseases (NIDDK) and the interstitial cystitis association (ICA) international research symposium october 19–20, 2000, Minneapolis, MN. Rev. Urol. 3, 146–151. 16985707 PMC1476046

[B94] Young R. H. (2009). Tumor-like lesions of the urinary bladder. Mod. Pathol. 22, S37–S52. 10.1038/modpathol.2008.201 19494852

