# Corrigendum: Inflammation and Tissue Remodeling in the Bladder and Urethra in Feline Interstitial Cystitis

**DOI:** 10.3389/fnsys.2018.00058

**Published:** 2018-11-08

**Authors:** F. Aura Kullmann, Bronagh M. McDonnell, Amanda S. Wolf-Johnston, Andrew M. Lynn, Daniel Giglio, Samuel E. Getchell, Wily G. Ruiz, Irina V. Zabbarova, Youko Ikeda, Anthony J. Kanai, James R. Roppolo, Sheldon I. Bastacky, Gerard Apodaca, C. A. Tony Buffington, Lori A. Birder

**Affiliations:** ^1^Department of Medicine, School of Medicine, University of Pittsburgh, Pittsburgh, PA, United States; ^2^Department of Pharmacology, The Sahlgrenska Academy, Göteborg University, Göteborg, Sweden; ^3^Department of Pharmacology and Chemical Biology, School of Medicine, University of Pittsburgh, Pittsburgh, PA, United States; ^4^Department of Pathology, School of Medicine, University of Pittsburgh, Pittsburgh, PA, United States; ^5^Department of Medicine and Epidemiology, University of California, Davis, Davis, CA, United States; ^6^Department of Veterinary Clinical Sciences, The Ohio State University, Columbus, OH, United States

**Keywords:** bladder, urethra, paraneurons, urothelium, von Brunn's nest

Daniel Giglio was not included as an author in the published article. The authors apologize for this error and state that this does not change the scientific conclusions of the article in any way. The original article has been updated.

## Author contributions

FAK and LAB: designed the study and wrote the manuscript. FAK, BMM, AML, DG, SEG, ASW-J, WGR, IVZ, YI, JRR, SIB and GA: designed, collected and analyzed data. CATB: diagnosed and characterized the cats, and provided them for this study. AJK and LAB: supervised personnel.

### Conflict of interest statement

The authors declare that the research was conducted in the absence of any commercial or financial relationships that could be construed as a potential conflict of interest.

